# The effect of *Macleaya cordata* extract on in vitro ruminal fermentation and methanogenesis

**DOI:** 10.1002/fsn3.2436

**Published:** 2021-07-03

**Authors:** Ze Zeng, Ping Sheng, Huaqi Zhang, Li He, Jiangli Huang, Dongsheng Wang, Ganbei Gui

**Affiliations:** ^1^ Key Open Laboratory of Chinese Veterinary Medicine of State Ethnic Affairs Commission Tongren Vocational and Technical College Tongren China; ^2^ Institute of Biological Resources Jiangxi Academy of Sciences Nanchang China

**Keywords:** in vitro, *Macleaya cordata* extract, methane production, rumen fermentation

## Abstract

Methane production is the main gas energy loss in ruminants and generates a powerful greenhouse gas that contributes to climate changes. *Macleaya cordata* is a plant commonly utilized additive in livestock diet as it contains various chemical compounds with beneficial health effects. This study aimed to explore the influence of *M. cordata* extract on in vitro methanogenesis and rumen fermentation. Three cannulated Jingjiang cattle were used as rumen fluid donors. The mixture of rumen fluid and a buffer (60 ml, 1:2 volume ratio) was treated with 6 different concentrations of *M. cordata* extract (0.01%, 0.11%, 0.21%, 0.31%, 0.41%, and 0.51%) and incubated for 12 and 24 hr. The control sample, with no addition of plant extract, was also analyzed. At each time point, pH, total gas, methane, dry matter (DM) digestibility, neutral detergent fiber (NDF), acid detergent fiber (ADF), ammonia nitrogen (NH_3_‐N), microbial protein (MCP), and volatile fatty acids (VFA) concentrations were determined. Total gas production decreased with increasing the amount of *M. cordata* extract at all time points. Methane production also decreased dose‐dependently with *M. cordata* extract after 3, 6, 9, and 12 hr of incubation, but increased after 24 hr. *M. cordata* extract decreased the concentration of NH_3_‐N and VFA, and the amount of acetic, propionic, and butyric acid. *M. cordata* extract decreased the MCP concentration after 12 hr, but its level returned to the initial value after 24 hr. Supplementation with 0.01, 0.11, and 0.21% of *M. cordata* extract did not affect the DM digestibility. However, supplementation with 0.31, 0.41, and 0.51% of *M. cordata* extract significantly decreased the DM digestibility. Supplementation with 0.11% of *M. cordata* extract effectively reduced methane production without affecting the DM digestion in vitro. However, its effect on in vivo methane production, rumen fermentation, and ruminant production requires further investigation.

## Graphical abstract

Methane production from ruminant not only wastes energy but also contributes to the greenhouse effect. This study found that the levels of *M*. *cordata* extract at 0.11% decreased methane production without affecting the DM disappearance. The results indicated that *M*. *cordata* extract have properties to decrease methanogenesis and may have the potential possibility for use as additives for ruminants.

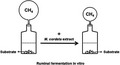

## 1 INTRODUCTION

The growing amount of greenhouse gases such as methane in the atmosphere poses a huge risk to global warming, as its warming potential is 25‐fold higher than carbon dioxide (Gill et al., 2010). Therefore, there is an urgent need for the reduction in methane in the atmosphere. The unique digestion metabolism of ruminants produces methane, and this process represents an energy loss as 2%–15% of dietary intake is utilized in methanogenesis (Ellis et al., 2007; Johnson & Johnson, 1995). Therefore, the reduction in methanogenesis in ruminants is an important goal for environment protection and the development of more efficient farming technologies.

*Macleaya cordata* is a medicinal plant native to China. It contains several active alkaloids such as sanguinarine and chelerythrine (Kosina et al., 2010; Shi et al., 2015). Due to potent antimicrobial, antifungal, anti‐inflammatory, and pesticidal activity, *M. cordata* is commonly used as a natural additive to livestock (swine, bovine, poultry, and fish) diet (Kantas et al., 2015; Niu et al., 2012; Zhang et al., 2013). Other studies have demonstrated that *M. cordata* promotes the growth of weaning pigs and broiler chickens (Kantas et al., 2015; Li, Zhang, et al., 2018). However, few studies focused on in vitro effect of *M. cordata* on ruminal fermentation metabolism and gas production. Therefore, this study aimed to explore the influence of *M. cordata* extract added in the livestock diet on in vitro fermentation parameters such as total gas production, methane production, dry matter (DM) disappearance, neutral detergent fiber (NDF) content, acid detergent fiber (ADF) content, ammonia nitrogen (NH_3_‐N) concentration, microbial protein (MCP) concentration, and volatile fatty acids (VFA) composition.

## 2 MATERIALS AND METHODS

### 2.1 Animal care

All experiments conducted in this study were done following the national legislation and good scientific practices, and the procedures were approved by the Animal Ethics Committee of the Jiangxi Academy of Sciences.

### 2.2 *Macleayacordata* extract

*Macleaya cordata* extract was obtained from Hunan Micolta Bioresource Inc. in China. The *M. cordata* extract is a solid powder with 40% sanguinarine and 20% chelerythrine and is stored in a dark and dry place.

### 2.3 *Invitro* fermentation design

The rumen fluid was taken from three cannulated Jingjiang cattle (250 ± 15 kg) that were provided with ad libitum sources of water, minerals, and vitamins. The cattle were fed with ryegrass and concentrated in the ratio of 60:40 twice daily at 09:00 and 17:00, equivalent to 2% of body weight. The rumen fluid was collected before morning feeding. The rumen fluid was stored in a bottle, previously kept warm and filled with O_2_‐free CO_2_ gas, carried to the laboratory, and filtered through four layers of cheesecloth before mixing with artificial saliva (1:2) maintained at 39℃ and under O_2_‐free N_2_ gas.

The following six dietary regimens were applied: control (CON), basal diet without *M. cordata* extract; treatment 1 (TM1), basal diet with 0.01% *M. cordata* extract; treatment 2 (TM2), basal diet with 0.11% *M. cordata* extract; treatment 3 (TM3), basal diet with 0.21% *M. cordata* extract; treatment 4 (TM4), basal diet with 0.31% *M. cordata* extract; treatment 5 (TM5), basal diet with 0.41% *M. cordata* extract; and treatment 6 (TM6), basal diet with 0.51% *M. cordata* extract (amount of *M. cordata* extract was relative to substrate). The rumen fluid‐buffer mixture (60 ml) was transferred anaerobically into a 150‐ml serum bottle containing 0.5 g of substrate (Table 1) and *M. cordata* extract, filled with O_2_‐free N_2_ gas, and then capped with a rubber stopper. The serum bottles were then incubated with shaking (100 rpm/min) at 39℃ for 12 hr and 24 hr. The in vitro fermentation experiment was a completely randomized block design with three runs and five replicates per run. Each run was conducted on separate weeks.

**TABLE 1:** Ingredient and chemical composition of incubation substrate

Item	Content
Ingredients (%)	
Ryegrass	50
Corn	34.8
Wheat bran	6.5
Soybean meal	6
Calcium hydrogen phosphate	0.9
Salt	0.5
Limestone	0.8
Vitamin–mineral premix^a^	0.5
Nutrient level (%)	
Dry matter	88.17
Combined net energy (MJ/kg)	6.13
Crude protein	15.01
Crude fat	4.36
Crude fiber	11.78
Calcium	0.77
Total phosphorus	0.43

^a^ The vitamin–mineral premix contains the following amounts of nutrients per kg of the diets: vitamin A, 300 KIU; vitamin D, 100 KIU; vitamin E, 1,200 mg; Fe, 4,000 mg; Cu, 600 mg; Zn, 2,500 mg; Mn, 3,000 mg; I, 60 mg; Co, 30 mg; and Se, 20 mg.

### 2.4 Gas production measurement and ruminal fermentation profiles

Upon completion of incubation, total gas production was quantified according to the method of Theodorou et al. (1994). A detachable pressure transducer and a digital readout voltmeter were used to measure the headspace gas pressure of fermenting cultures after removing serum bottles from a shaking incubator. The transducer was connected to the inlet of a disposable Luer‐lock three‐way stopcock in order to quantify the total gas production in this system. Gas pressure in the headspace was read from the display unit after insertion of the hypodermic syringe needle through the rubber stopper above the culture medium. The amount of methane in a headspace gas above the culture medium was analyzed using gas chromatography (GC‐2010, Shimadzu). The culture medium was analyzed for pH (FE‐28, Mettle‐Toledo), VFA concentration, NH_3_‐N, and MCP concentration. The VFA analysis was performed with a gas chromatography (GC‐2010, Shimadzu) as described method by Stewart and Duncan (Stewart & Duncan, 1985). The UV/Vis spectroscopy (Model 680, Bio‐Rad laboratories) was applied for the quantification of NH_3_‐N and MCP following the procedure described by Wang et al. (2008) and Wang (2003). The rate of DM disappearance was determined using the nylon bag digestion method in vitro. After incubation, the nylon bags with substrate were rinsed with cold water until the water ran clear, dried at 60℃ until constant weight, weighed, and subsequently analyzed for DM digestibility.

### 2.5 Statistical analysis

The data analysis was performed using one‐way analysis of variance (ANOVA) using SPSS version 16.0 software. To test the effect of *M. cordata* extract on total gas production, methane production, pH, DM disappearance, and VFA profiles, the results were compared with control experiment, and the statistical significance between the results was obtained by applying Duncan's multiple comparison tests. A *p* < .05 was considered to be statistically significant.

## 3 RESULTS

### 3.1 Gas production kinetics

The effects of *M. cordata* extract dose on total gas production and methane emission are shown in Table 2. Total gas production in samples supplemented with 0.11%, 0.21%, 0.31%, 0.41%, and 0.51% of *M. cordata* extract was lower than the value for CON (*p* < .05). The methane emission in samples with added 0.11%, 0.21%, 0.31%, 0.41%, and 0.51% of *M. cordata* extract was significantly lower compared with CON after 3, 6, 9, and 12 hr (*p* < .05). However, the samples supplemented with 0.11%, 0.21%, 0.31%, and 0.41% of *M. cordata* extract and incubated for 24 hr exhibited significantly higher methane emission compared with the CON sample (*p* < .05).

**TABLE 2:** The effect of *M. cordata* extract on gas and methane emission according to incubation time

Incubation (hr)	Treatment^a^							*SEM*	*p*
	CON	TM1	TM2	TM3	TM4	TM5	TM6		
Gas emission (ml/g DM)									
3 hr	19.19	17.06	10.66**	9.95**	9.24**	8.53**	8.53**	2.26	<.001
6 hr	67.29	60.89	44.54**	43.12**	40.99**	34.59**	33.88**	3.46	<.001
9 hr	144.52	134.57	96.19**	94.06**	87.66**	71.31**	68.47**	5.39	<.001
12 hr	211.33	200.67	161.58**	155.89**	147.36**	119.64**	115.38**	7.12	<.001
24 hr	319.37	316.53	306.57	303.02	299.47*	271.04**	256.11**	9.15	<.001
Methane emission (ml/g DM)									
3 hr	3.38	3.14	1.92**	1.89**	1.87**	1.79**	1.63**	0.12	<.001
6 hr	4.20	3.92	2.19**	2.15**	2.10**	1.96**	1.79**	0.14	<.001
9 hr	5.94	5.47	2.58**	2.49**	2.37**	2.13**	1.93**	0.24	<.001
12 hr	7.20	6.62	3.57**	3.40**	3.17**	2.55**	2.26**	0.29	<.001
24 hr	18.45	18.48	21.24**	21.79**	21.82**	20.81**	18.18	0.66	<.001

Abbreviations: DM, dry matter; *SEM*, standard error of the mean.

^a^ The following dietary treatments were applied: CON, basal diet (without *M. cordata* extract); TM1, CON + 0.01% *M. cordata*; TM2, CON + 0.11% *M. cordata*; TM3, CON + 0.21% *M. cordata*; TM4, CON + 0.31% *M. cordata*; TM5, CON + 0.41% *M. cordata*; and TM6, CON + 0.51% *M. cordata* (amount of extract was relative to substrate).

* *p* < .05,

** *p* < .01 relative to CON.

### 3.2 In vitro ruminal fermentation characteristics

The effects of *M. cordata* extract dose on pH and DM disappearance are shown in Table 3. The pH of the fermentation medium at 12‐hr and 24‐hr incubation was significantly affected by *M. cordata* extract. After 12‐hr incubation, pH of samples supplemented with 0.11%, 0.21%, 0.31%, 0.41%, and 0.51% of *M. cordata* extract increased (*p* < .05), while 24‐hr incubation with 0.21%, 0.31%, 0.41%, and 0.51% of *M. cordata* extract significantly lowered the pH of a sample (*p* < .05). The addition of lower levels of *M. cordata* extract (0.01%, 0.11%, and 0.21%) did not significantly affect the rate of DM disappearance (*p* > .05). However, a higher amount of *M. cordata* extract (0.31%, 0.41%, and 0.51%) significantly decreased the rate of DM disappearance after 12 hr and 24 hr of incubation (*p* < .05).

**TABLE 3:** The effect of *M. cordata* extract on pH, dry matter (DM) disappearance, ammonia nitrogen (NH_3_‐N), microbial protein (MCP), neutral detergent fiber (NDF), and acid detergent fiber (ADF) according to incubation time

Incubation (hr)	Treatment^a^							*SEM*	*p*
	CON	TM1	TM2	TM3	TM4	TM5	TM6		
pH									
12 hr	6.85	6.87	6.89*	6.90*	6.90*	6.93**	6.91**	0.02	.007
24 hr	6.71	6.68	6.69	6.67*	6.65**	6.67*	6.67*	0.02	.017
DM disappearance (%)									
12 hr	48.48	48.24	48.04	47.96	44.36**	43.40**	39.84**	1.01	<.001
24 hr	60.61	60.37	59.53	59.37	57.73**	54.85**	50.89**	0.65	<.001
NH_3_‐N concentration (mg/l)									
12 hr	112.30	110.66	98.53**	92.99**	88.10**	82.89**	75.80**	2.30	<.001
24 hr	161.89	157.45	148.68**	146.69**	144.77**	137.21**	134.75**	3.23	<.001
MCP concentration (mg/100 ml)									
12 hr	70.11	69.21	55.80**	53.80**	54.35**	53.27**	52.02**	1.69	<.001
24 hr	72.69	73.31	76.61	76.01	75.34	75.18	72.56	1.95	.238
NDF (%)									
12 hr	59.93	59.83	60.56	60.80	61.10	61.36	61.57	0.83	.307
24 hr	57.08	57.36	56.77	56.36	56.46	56.08	56.86	1.24	.951
ADF (%)									
12 hr	38.10	38.50	38.74	39.03	39.54	39.36	39.81	0.86	.463
24 hr	31.61	31.85	31.02	30.31	30.63	30.37	30.94	0.73	.313

Abbreviations: ADF, acid detergent fiber; DM, dry matter; MCP, microbial protein; NDF, neutral detergent fiber; NH_3_‐N, ammonia nitrogen; *SEM*, standard error of the mean.

^a^ The following dietary treatments were applied: CON, basal diet (without *M. cordata* extract); TM1, CON + 0.01% *M. cordata*; TM2, CON + 0.11% *M. cordata*; TM3, CON + 0.21% *M. cordata*; TM4, CON + 0.31% *M. cordata*; TM5, CON + 0.41% *M. cordata*; and TM6, CON + 0.51% *M. cordata* (amount of extract was relative to substrate).

* *p* < .05,

** *p* < .01 relative to CON.

The influence of *M. cordata* extract on NH_3_‐N, MCP, NDF, and ADF in rumen fluid is shown in Table 3. The NH_3_‐N concentration significantly decreased with the addition of 0.11%, 0.21%, 0.31%, 0.41%, and 0.51% of *M. cordata* extract at 12 hr and 24 hr of incubation (*p* < .05). The MCP concentration decreased upon supplementation with 0.11%, 0.21%, 0.31%, 0.41%, and 0.51% of *M. cordata* extract after 12 hr of incubation (*p* < .05), but remained unchanged after 24 hr of incubation (*p* > .05). The content of NDF and ADF was unaffected by *M. cordata* extract at all time points (*p* > .05).

### 3.3 Volatile fatty acid profile and acetic acid/propionic acid ratio

The outcome of supplementation with *M. cordata* extract on the content of VFA in rumen fluid such as acetic, propionic, butyric acid, and acetic acid/propionic acid ratio (A/P) is shown in Table 4. The total VFA concentration, and the level of acetic, propionic, and butyric acid were significantly reduced upon 12‐hr incubation with *M. cordata* extract (*p* < .05).

**TABLE 4:** The effect of *M. cordata* extract on total volatile fatty acids (VFA), acetic acid, propionic acid, butyric acid, and acetic acid/ propionic acid ratio (A/P) according to incubation time

Incubation (hr)	Treatment^a^							*SEM*	*p*
	CON	TM1	TM2	TM3	TM4	TM5	TM6		
Total VFA concentration (mg/ml)									
12 hr	4.30	4.05	3.85**	3.64**	3.81**	3.57**	3.39**	0.13	<.001
24 hr	5.16	5.02	5.06	5.11	4.95	4.78**	4.99	0.13	.110
Acetic acid concentration (mg/ml)									
12 hr	2.79	2.63	2.50**	2.37**	2.48**	2.32**	2.20**	0.08	<.001
24 hr	3.35	3.24	3.28	3.29	3.20	3.10**	3.22	0.09	.126
Propionic acid concentration (mg/ml)									
12 hr	1.04	0.99	0.94*	0.88**	0.93**	0.90**	0.81**	0.04	.001
24 hr	1.23	1.21	1.21	1.25	1.22	1.17	1.24	0.04	.464
Butyric acid concentration (mg/ml)									
12 hr	0.46	0.43	0.41*	0.39**	0.40*	0.35**	0.36**	0.02	.005
24 hr	0.59	0.57	0.57	0.57	0.53**	0.51**	0.53**	0.02	.008
A/P ratio									
12 hr	2.69	2.67	2.66	2.69	2.68	2.59*	2.71	0.04	.144
24 hr	2.73	2.69	2.72	2.64	2.63*	2.66	2.60*	0.05	.131

Abbreviations: A/P, acetic acid/ propionic acid; *SEM*, standard error of the mean; VFA, volatile fatty acids.

^a^ The following dietary treatments were applied: CON, basal diet (without *M. cordata* extract); TM1, CON + 0.01% *M. cordata*; TM2, CON + 0.11% *M. cordata*; TM3, CON + 0.21% *M. cordata*; TM4, CON + 0.31% *M. cordata*; TM5, CON + 0.41% *M. cordata*; and TM6, CON + 0.51% *M. cordata* (amount of extract was relative to substrate).

* *p* < .05,

** *p* < .01 relative to CON.

## 4 DISCUSSION

Alkaloids have received interest as promising alternatives to antibiotics in ruminant feeding due to their ability to reduce methane production (Cristina et al., 2017). *Macleaya cordata* is a traditional medicinal herb from China with several biologically active alkaloids including sanguinarine and chelerythrine (Kosina et al., 2010; Shi, Liu, Shao, We, & Lin, 2015).

In vitro experiments revealed a positive correlation between the gas production and digestibility of organic matter (Menke & Steingass, 1988). The total gas production at all time points was lower compared with control sample, suggesting that *M. cordata* extract decelerates diet digestion by rumen microorganisms. Previous studies reported that supplementation with *M. cordata* extract decreased gas production in the rumen fermentation of Hu sheep in vitro (Zhang et al., 2017), which is consistent with the results of this study. Moreover, the extracts enriched with alkaloids are recognized as a promising feed additive to reduce gas production during ruminal fermentation (Santos et al., 2013).

The addition of *M. cordata* extract to the diet significantly decreased methane production after 3, 6, 9, and 12 hr of incubation, but the gas production increased after prolonged incubation (24 hr). This trend might be explained by the inhibitory effect of *M. cordata* extract on methanogens that was most pronounced at the beginning, and gradually disappeared in the next 12 hr, After 12 hr, the activity of methanogens increased rapidly, which resulted in the increased methane production after 24 hr. The inhibitory effect of *M. cordata* extract on methane production is reported for in vitro rumen fermentation model of Hu sheep (Zhang et al., 2017). In addition, previous studies confirmed the potential of alkaloid‐enriched extract to reduce methanogenesis (Cristina et al., 2017; Joch et al., 2018). The analysis of VFA profiles provides additional insights into the mechanism of how *M. cordata* extract inhibits methane production. The acetic and butyric acids are natural substrates for methane formation (Kumar et al., 2009). The reduced amount of acetic and butyric acids may be another mechanism responsible for the decreased methane production. However, additional studies on the effects of *M. cordata* extract on ruminal bacteria and the archaeal community are still needed to clarify the actual mechanisms of action.

NH_3_‐N is an important product of rumen digestion and metabolism, and it is also the raw material for most microorganisms to synthesize bacterial protein. The content of NH_3_‐N directly reflects the fermentation process in the rumen environment (Benchaar et al., 2008). The results of the present study revealed that the addition of *M. cordata* extract significantly reduces the concentration of NH_3_‐N, which is consistent with the literature (Zhang et al., 2017). This phenomenon might be explained by the inhibitory activity of *M. cordata* extract on the microbial utilization of feed protein, resulting in the decreased amount of NH_3_‐N. MCP is an important indicator of rumen fermentation as it reflects the number of bacteria, protozoa, fungi, and other microorganisms in the rumen (Li et al., 2018). The present study showed that the addition of *M. cordata* extract reduces the ruminal MCP concentration after 12‐hr incubation, while the MCP returns close to the initial value after 24 hr. This trend is explained by the inhibitory activity of *M. cordata* extract on rumen microorganisms, which gradually disappeared after 12 hr and allowed the growth and recovery of the microorganisms after 24 hr.

VFA is the main source of energy in ruminants, and their content and composition directly reflect the level of metabolic activity in the rumen (Lee et al., 2018a, 2018b). In the present study, the addition of *M. cordata* extract steadily decreased the total VFA, acetic acid, propionic acid and butyric acid concentration, and A/P ratio. This result is not consistent with the literature (Zhang et al., 2017), and the observed discrepancy could be ascribed to different dosage regimens used in two studies. Moreover, the results showed that *M. cordata* extract had a significant effect on pH. Nevertheless, the pH value remains within the range 6.37–7.35, which is an appropriate pH range for cellulose digestion (6.0–6.8), protein synthesis (6.3–7.4), proteolytic activity (6.5–7.0), VFA production (6.0–6.6), and ruminal microbial activity (5.8–7.2; Hiltner & Dehority, 1983; McCullough et al., 1969). Compared with the control group, the DM disappearance did not change significantly when *M. cordata* extract was added at 0.01%, 0.11%, and 0.21%, while DM digestibility decreased when the dosage was 0.31%, 0.41%, and 0.51%. The results of our study suggest that the supplementation of the diet with more than 0.31% of *M. cordata* extract might reduce the rumen fermentation and DM digestion.

## 5 CONCLUSION

The present study shows that the addition of 0.11% *M. cordata* extract in diet decreases methane production, VFA, NH_3_‐N, and MCP production, without affecting the DM digestibility. These results suggest that supplementation with 0.11% of *M. cordata* extract decreases methanogenesis without negative impact on ruminal fermentation and could be used as a potential additive for ruminants. Additional research is required to evaluate the effects of *M. cordata* extract on in vivo ruminal methane production, fermentation, and microbiome, which is required for this extract to be used as a food additive in ruminant production.

## CONFLICT OF INTEREST

The authors confirm that there is no conflict of interests regarding this paper.

## AUTHOR CONTRIBUTIONS

**ze zeng:** Conceptualization (equal); Data curation (lead); Funding acquisition (equal); Project administration (equal); Writing‐original draft (lead); Writing‐review & editing (equal). **Ping Sheng:** Conceptualization (equal); Data curation (equal); Funding acquisition (equal); Project administration (equal); Writing‐original draft (equal); Writing‐review & editing (equal). **Huaqi Zhang:** Conceptualization (lead); Funding acquisition (equal); Methodology (equal); Project administration (equal); Writing‐review & editing (equal). **Li He:** Conceptualization (supporting); Methodology (supporting); Resources (equal). **Jiangli Huang:** Conceptualization (supporting); Resources (supporting). **Dongsheng Wang:** Conceptualization (supporting); Resources (supporting). **Ganbei Gui:** Conceptualization (supporting); Resources (supporting).

## ETHICAL APPROVAL

The study protocol was approved by the Animal Ethics Committee of the Jiangxi Academy of Sciences.

## Data Availability

The data that support the findings of this study are available from the corresponding author upon reasonable request.
